# Results of meniscal injuries repair using different arthroscopic techniques

**DOI:** 10.1051/sicotj/2020030

**Published:** 2020-10-07

**Authors:** Mohamed Abdel Tawab Abdallah, Hatem G. Said, Eslam Karam Allah Ramadan, Mohamed Abd El-Radi, Maher A. El-Assal

**Affiliations:** 1 Resident of Orthopedic Surgery, Faculty of Medicine, Assiut University 71515 Assiut Egypt; 2 Professor of Orthopedics and Traumatology, Faculty of Medicine, Assiut University 71515 Assiut Egypt; 3 Lecturer of Orthopedics and Traumatology, Faculty of Medicine, Assiut University 71515 Assiut Egypt

**Keywords:** Knee, Meniscal tear, Arthroscopic, Repair, Meniscectomy

## Abstract

*Aim*: Evaluation of clinical and radiological outcomes following meniscal repair using different arthroscopic techniques for all meniscal tears amenable for repair. *Methods*: Sixty-one patients were involved in a prospective study; all cases presented with meniscal tears underwent arthroscopic meniscal repair from December 2016 to December 2017. Outcomes involved the site of tear, the repair technique, and associated injuries. The International Knee Documentation Committee Score (IKDC) and Tegner Lysholm Knee Score were used to analyze the clinical and functional outcomes postoperatively. *Results*: Of the 61 patients, 50 patients (81.9%) had meniscal tear associated with isolated ligamentous ACL injury, 6 cases had corrective osteotomy with ACL reconstruction to correct concomitant genu varus, 2 cases (3.3%) had meniscal tear associated with isolated ligamentous PCL injury, and 9 patients (14.8%) presented with isolated meniscal tear; IKDC was preoperatively (44.52 ± 8.79), postoperatively at 6 months (90.97 ± 6.75) and at 12 months (92.27 ± 2.68) with *P*-value (0.001). Tegner Lysholm score was preoperatively (52.16 ± 12.22), postoperatively at 6 months (88.03 ± 6.84) and at 12 months (93.26 ± 2.95) with *P*-value (0.001). Fifty eight patients (95.1%) had no postoperative symptoms at 6 and 12 months’ follow-up. The remaining 3 cases (4.9%) underwent partial meniscectomy due to persistent postoperative clinical symptoms with no signs of healing in MRI. *Conclusions*: Our study concluded that arthroscopic meniscal repair is an effective way in the management of meniscal tears regarding clinical and functional outcomes.

## Introduction

The meniscus plays an important role in knee function by providing structural and biomechanical roles in congruence, joint load distribution and bearing, stability, lubrication, proprioception, and nutrition [1]. The main cause of surgical management for meniscal tears is to relieve the corresponding symptoms like pain, clicking, to facilitate the daily living activity, and prevent early degenerative diseases of the knee joint [2].

Henning et al. suggested that not all meniscal tears need a surgical intervention. Many tears cause no clinical symptoms or functional problems; so these types do not need a surgical treatment because they will remain asymptomatic or heal spontaneously [3].

Nowadays, most of meniscal tears are treated by arthroscopic repair; however, common criteria of the meniscal tear that are amenable for repair include: a tear within 3–4 mm of the meniscocapsular junction or the peripheral 10% to 30% of the meniscus, complete vertical tear >10 mm, a tear without secondary degenerative diseases or deformity, a symptomatic tear in young patients, and a tear without multiligamentous injury or stable knee. When these criteria are present, formal repair using a variety of methods should be conducted [4].

Techniques of meniscal repair have been developed and modified over the years. The aim of using a combination of techniques in repair is to achieve the optimal stability to the meniscal tear. The standard precautions in meniscal repair are to remove any loose or frayed fragments and promote healing by rasping the opposing edges [2].

The aim of our study was to evaluate and analyze the outcome of arthroscopic meniscal repair at arthroscopy unit in our hospital.

## Material and methodology

A prospective non-randomized case series study was carried out in Arthroscopy and sports injury unit in our hospital from December 2016 to December 2017. Diagnosis was confirmed through clinical history, physical examination, and radiological assessment including plain radiograph and magnetic resonance imaging (MRI).

All patients were evaluated preoperatively and postoperatively by the International Knee Documentation Committee Score (IKDC) [5] and Tegner Lysholm Knee Score [6].

A sample of 30 patients were followed up by MRI at 6 months postoperatively. Meniscal repair healing assessment using MRI was classified according to Crues et al. [7] to 4 Grades: Grade 0 (normal meniscus), Grade I shows focus intrameniscal signal intensity, Grade II represents as linear or wedge-shaped intrameniscal signal intensity, and Grade III represents the signal intensity extending to the articular surface. The repair was considered healed in case of Grade 0, I, or II. A non-healed repair was diagnosed if signal intensity extended to the articular surface (Grade III).

Patients with age range from 15 to 45 years, repairable meniscal tears with or without isolated ligamentous injury even with concomitant genu varus during 6 months of trauma, tear in red or red-white zone, bucket-handle tear, radial tear, longitudinal tear, horizontal tear, oblique tear, root tear, and all standard techniques of repair were involved in our study.

Patients with age range <15 years or >45 years, had complex tear, root tear, degenerative tear, tear in white zone, associated multiligamentous knee injury, and degenerative osteoarthritis were excluded.

### Surgical techniques

Patients were positioned supine on an ordinary table after anesthesia (spinal or general). A tourniquet was positioned at the proximal of the thigh. A support under the lateral side of the thigh was used. Standard knee portals (anteromedial and anterolateral portals) were used in all cases. Additional portals were used regarding different techniques.

### Instruments

We used curved and straight suture Lasso (Biomet), Suture Passer (Arthrex), FasT-Fix (Smith and Nephew), Meniscal Cinch (Arthrex), MaxFire (Biomet), and sometimes a straight Cannula. We used non-absorbable sutures such as Maxbraid suture (Zimmer Biomet), Fiberwire (Arthrex), and Orthocord suture (Depuy mitek).

### Inside-out technique

**Figure 1:**
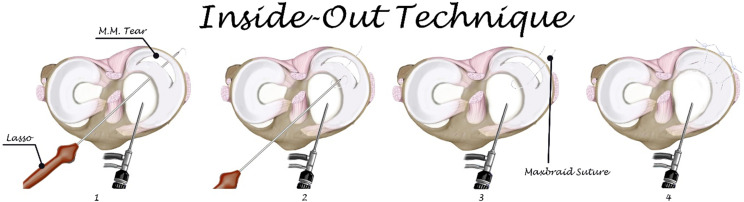
Steps of Inside-out technique.

After arthroscopic debridement and preparing the site of tear, the knee was placed in 20° to 30° of flexion and posteromedial incision was made in case of medial meniscus (MM) tear. Two-to-three-cm longitudinal incision was made posterior to the medial collateral ligament through the sartorial fascia and anterior to the semimembranosus deep to medial head of gastrocnemius muscle without violating the capsule to catch suture lasso from inside to outside the knee. The knee was placed in 90° of flexion in case of lateral meniscal (LM) tear; 2–3-cm longitudinal incision was made posterior to the lateral collateral ligament and anterior to the biceps femoris tendon through iliotibial band, deep and anterior to the lateral head of the gastrocnemius without violating the capsule. The suture lasso was loaded by non-absorbable suture and directed toward the tear, the lasso passed through the meniscus out of the capsule and superior to the tear. The suture lasso was directed again to the meniscus, below the tear, out of the joint capsule and loaded with the other end of the suture. Suture was tied over the joint capsule. Arthroscopic visualization was used during suturing to ensure the optimal tension as shown in Figure 1.

### Outside-in technique

**Figure 2:**
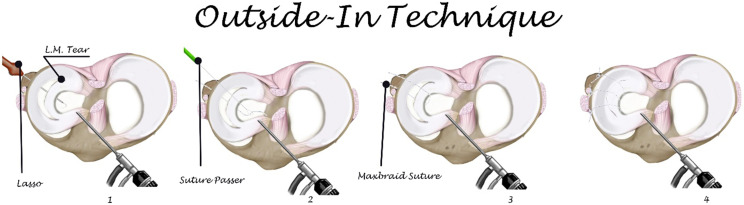
Steps of Outside-in technique.

After debridement and preparing the site of tear, we used a needle to identify the location of tear from outside the knee; a small incision was made at the joint line and the site of tear without violating the capsule. A straight suture Lasso loaded by non-absorbable suture was inserted from outside the knee, through the peripheral meniscal rim and below the tear unloaded one end of the suture. Another lasso inserted from outside the knee and superior to the tear was loaded by wire loop. A suture passer was inserted in the ipsilateral portal drive at the end of the non-absorbable suture to the wire loop. The suture Lasso was carefully removed from the joint hold at the end of the non-absorbable suture. Both ends of the non-absorbable suture were brought together subcutaneously and tied over the capsule under arthroscopic visualization to guarantee the optimal tension on the knots as shown in Figure 2.

### All inside technique

Meniscal Cinch (Arthrex) was the most used device in our study. We used the device through the anteromedial portal for MM tear and anterolateral portal for LM tear. After preparing the tear site, we located the entry point and measured the distance between the entry point and the capsule using graduated tip of the Cinch cannula. The first implant was advanced through the meniscus at the edge of the tear. The tip of the cannula was moved to the second insertion point over the meniscus. The second implant was advanced through the meniscus until the trocar handle made contact with the depth stop and the cannula reset on the surface. The Meniscal Cinch was removed from the joint. The external suture was tensioned and pushed the knot using the knot pusher. The knot was tied under arthroscopic visualization.

### Postoperative rehabilitation

Pain and edema were managed using ice, elevation, and medication. The postoperative rehabilitation was divided into four protocols according to the type of the operative procedure. Partial weight bearing using crutches from day one postoperative was allowed, active knee flexion and extension with range of motion (0°–90°) gradually for 6 weeks, strengthening and stretching exercises for quadriceps and calf muscles, and full extension ACL brace was used for 6 weeks in case of isolated meniscal injury and in association with ACL reconstruction. The previous protocol was used but with non-weight bearing for 6 weeks in case of genu varum correction.

Non-weight bearing was allowed for 6 weeks, passive knee flexion and extension with range of motion (0°–90°) gradually for 6 weeks, strengthening exercises for hamstrings and quadriceps at 6 weeks, and ACL brace was used in case of meniscal repair associated with PCL reconstruction.

### Postoperative follow-up

Check-up of all patients at 2 weeks postoperatively was for stitch removal, 6 weeks for knee examination, and start of full-weight bearing for genu varum correction and PCL reconstruction cases. Six months’ follow-up was for clinical evaluation by scoring systems (IKDC and Lysholm) and the absence of persistent symptoms including joint line tenderness, swelling, clicking, and negative McMurray test (Barrett’s criteria [8]). A sample of 30 patients were assessed radiologically by MRI at 6 months postoperatively. All patients were assessed at 1 year postoperatively by knee scoring system.

### Failure of repair

Presence of pain not relieved by analgesics, clicking, tenderness of joint line, positive McMurray test, low scoring system, and a persistent tear in MRI (grade III) were considered as failure of repair.

### Statistical analysis

Data entry and analysis were done using SPSS (Statistical Package for Social Science) version 19. Data were reviewed as number, percentage, mean, and standard deviation. Paired samples *t*-test was done to compare quantitative data between preoperative and postoperative. *P*-value was considered statistically significant when it was <0.05.

## Results

**Figure 3:**
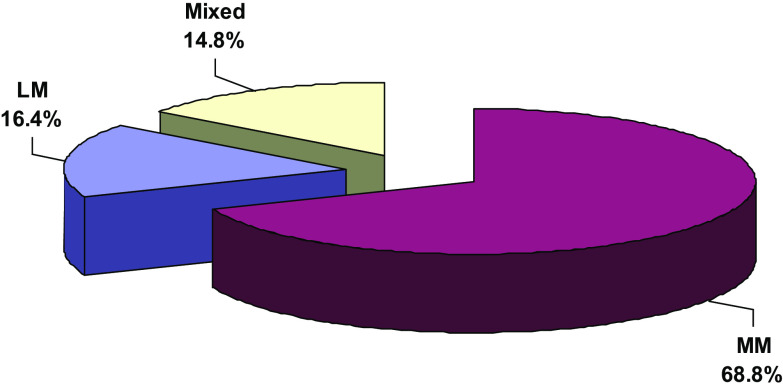
Percentage of meniscal tear between medial and lateral menisci.

A total of 61 patients were involved with age range from 16 to 42 years. Sports injury was the most common cause of trauma in our study which represented as 55.7%, followed by falling down stairs (26.2%). There were a total of 42 cases of MM tear (68.8%), 10 LM tears (16.4%), and 9 mixed MM and LM tear (14.8%) as described in Figure 3.

Fifty-two patients (85.2%) had meniscal tear associated with isolated ligamentous injury (ACL or PCL). Fifty cases (81.9%) had ACL tear and underwent ACL reconstruction. Six cases had genu varum deformity which was corrected using high tibial open wedge osteotomy and ACL reconstruction. Two cases (3.3%) presented with PCL tear and underwent PCL reconstruction. There were 9 cases (14.8%) presented with isolated meniscal tear.

We categorized the repair according to the technique (outside-in, inside-out, all-inside, and combined techniques), The most used technique was Inside-out technique which represents 62.3% of all cases as shown in Table 1.

**Table 1:** Percentage of techniques used for meniscal repair.

Technique	No. (*n* = 61)	%
All inside	5	8.2
Inside-out	38	62.3
Outside-in	13	21.3
Combined	5	8.2

**Figure 4:**
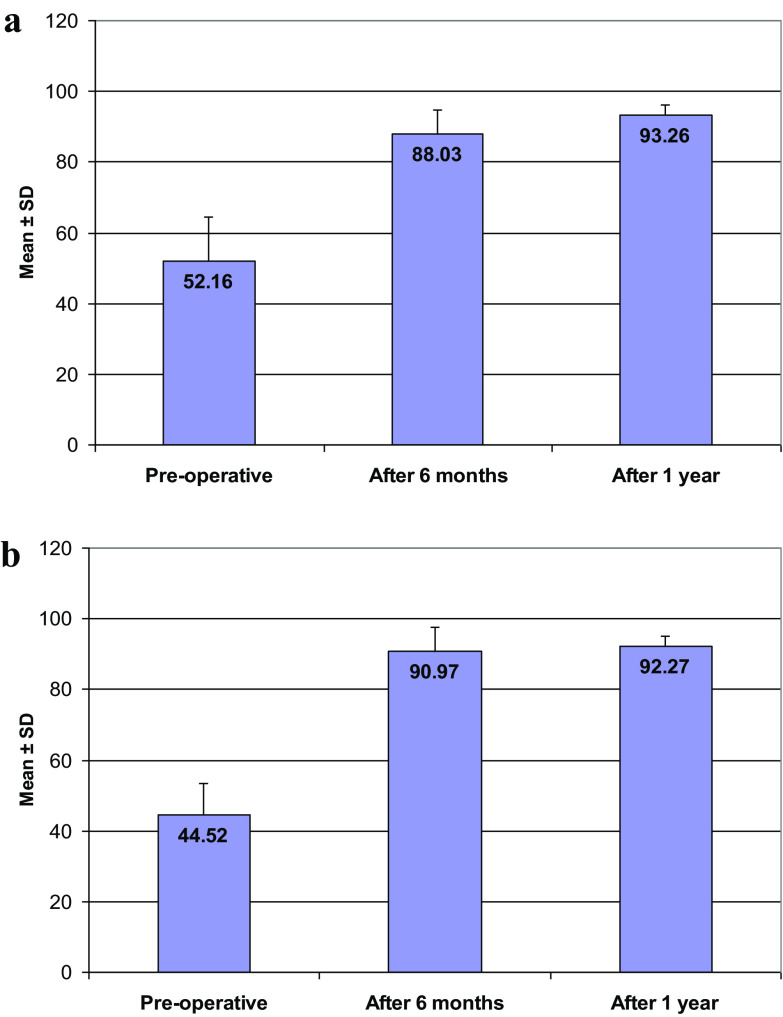
**(**a) Tegner Lysholm knee score. (b) IKDC score.

All patients were assessed postoperatively by IKDC at 6 months and at 1 year with mean ± SD of (90.97 ± 6.75) and (92.27 ± 2.68), respectively with *P*-value (0.001). Tegner Lysholm Knee Score mean ± SD at 6 months was (88.03 ± 6.84) and (93.26 ± 2.95) at 1 year with *P*-value (0.001) as described in Table 2 and Figure 4.

**Table 2:** IKDC and Tegner Lysholm Knee Score pre- and postoperatively.

	Preoperative	6 months postoperative	1 year postoperative	*P*-value^1^	*P*-value^2^
IKDC score					
Mean ± SD	44.52 ± 8.79	90.97 ± 6.75	92.27 ± 2.68		
Range	27.6–78.2	63.2–98.9	90.8–98.9		
Tegner Lysholm Knee Score					
Mean ± SD	52.16 ± 12.22	88.03 ± 6.84	93.26 ± 2.95	0.001*	0.001*
Range	27.0–95.0	67.0–99.0	86.0–99.0		

* *P*-Value^1^ and *P*-Value^2^ are the values at 6 months and 1 year postoperatively, respectively.

Patients who had ACL reconstruction and meniscal repair (50 cases) were assessed postoperatively by IKDC at 6 months and at 1 year with mean ± SD of (91.15 ± 5.96) and (95.05 ± 2.66), respectively, and were compared to isolated meniscal repair (9 cases) who were assessed postoperatively by IKDC at 6 months and at 1 year with mean ± SD of (89.96 ± 10.67) and (96.56 ± 2.55), respectively, with *P*-value (0.628) at 6 months and with *P*-value (0.120) at 1 year. Tegner Lysholm Knee Score in cases of ACL reconstruction and meniscal repair mean ± SD at 6 months was (88.00 ± 6.43) and (93.15 ± 2.97), respectively, at 1 year and were compared to isolated meniscal repair (9 cases) assessed by IKDC at 6 months and at 1 year with mean ± SD of (88.22 ± 9.35) and (93.89 ± 2.93), respectively, with *P*-value (0.959) at 6 months and with *P*-value (0.495) at 1 year.

We had 58 (95.1%) non-complicated meniscal repair clinically according to Barrett’s criteria and scoring system. However, there were 3 cases (4.9%) with persistent pain, clicking, positive McMurray test, low scoring system, and MRI grade III signal intensity according to the classification used by Crues et al. [7] within 6 months postoperatively. Those 3 cases underwent revision partial meniscetomy.

MRI evaluation of 30 patients at 6 months postoperatively showed, 16 cases (53.3%) classified as Grade I, Grade II signals and 14 cases (46.7%) classified as Grade III signal (non-healed repair) according to the classification used by Crues et al. [7].

Based on clinical evaluation of those 30 patients, 27 cases (90%) showed no postoperative complication when detected at 6 and 12 months’ follow-up. Three cases (10%) had failure of repair as shown in Table 3.

**Table 3:** Clinical and radiological evaluation of 30 patients who had MRI at 6 months postoperatively.

Evaluation	Healed	Non-healed
Radiological evaluation (MRI)	16 (53.3%)	14 (46.7%)
Clinical evaluation (knee scoring system)	27 (90%)	3 (10%)

We correlated clinical and radiological outcomes of patients who had MRI follow-up at 6 months (30 patients). Eleven patients (36.6%) were classified on radiological evaluation as non-healed repair despite having no clinical symptoms. Three cases (10.1%) presented with MRI Grade III signal intensity according to the classification used by Crues et al. [7] within 6 months postoperatively and persistent clinical Symptoms and underwent revision partial meniscetomy.

## Discussion

Meniscal repair is better than meniscectomy, if the tear is amenable for repair, as meniscus-deficient knees are at a significant risk of developing osteoarthritis (OA) and other degenerative diseases [9]. The aim of meniscal repair is to maintain maximal integrity of the meniscus which would allow us to prevent the symptoms that affect the quality of life associated with osteoarthritis, which is accelerated with loss of the meniscus.

In this study, medial meniscal tears were of higher frequency (68.8%) than lateral tears (16.4%). We thought that the differences in medial or lateral tear rate were supposedly due to the differences in lower limb alignment or due to the different mechanisms of injury. In contrast, Nikolić et al. [10] reported that the rate of lateral meniscal tear was higher than medial tear with an incidence of 72% in his study on 66 patients presented with acute ACL tear.

In contrast, our results showed that the most used technique of meniscal repair was inside-out technique (62.3%) and we did not find any relationship between the technique of meniscal repair and failure rate. We also found that a lot of repairable meniscal tears were associated with isolated ligamentous injury, mainly ACL (81.9%). There was an insignificant difference in comparing the results of meniscal repair in isolated meniscal tear and tear associated with ACL in pain satisfaction and activities of daily living in our study due to the presence of a small number of cases who had isolated meniscal tear.

According to Scott et al. [11], the inside-out technique of repair was the gold standard technique used for the management of 260 patients complaining of meniscal tear enrolled in their study, also study of Gulamhussein et al. [12] on 60 patients with isolated meniscal tear that underwent meniscal repair concluded that using both techniques (all-inside and inside out) was an effective method of treating young patients. Although they reported that the patients looked to improve by 12 months with regard to symptoms and daily living activities.

According to our study, 6-month MRI follow-up reported 11 patients (36.6%) with grade III signal presented without any clinical symptoms interfering with activity of daily living. We concluded that MRI signals of the meniscus after meniscal repair remaining occur within first 6 months postoperatively.

Willinger et al. [13] reported on 35 acute meniscal repair cases comparing the clinical outcome using clinical examination (Barrett criteria) and outcome scores (IKDC, KOOS and Lysholm Score) with radiological (MRI) outcome at several times postoperatively, which found that 64.3% healed according to clinical outcome and 55.9% with complete healing according to MRI at 6 months’ follow-up, they also reported that 44.1% of the repairs still did not heal completely in the radiological assessment.

Our MRI assessment of 30 cases at 6 months’ follow-up showed that 14 cases (46.7%) presented with non-healed meniscal repair according to MRI; however, 3 cases (10%) presented with clinical symptoms. These outcomes are similar to those of Pujol et al. [14] who reported in their study 58% of completely healed, 24% of partially healed, and 18% of failed repair cases after 6 months’ follow-up. We suspected that, this may be due to a continuous healing process which showed a lower rate of healed meniscus in near follow-up examinations, but the rate would improve over time.

Our failure rate was 4.9%. We did not find any role of age, site, type, and size of tear in our failure rates. In contrast, study of Gulamhussein et al. [12] showed that the failure rate was 23.3% with many patients with complications with re-tear revised by partial or total meniscectomy. It can be postulated that the differences in the failure rate was due to the presence of a large number of cases that had associated ligamentous injury (ACL or PCL) which enhances the healing of meniscal repair, consistent with the study of Scott et al. [11], which reported that the healing rate of meniscal repair in the presence of ACL reconstruction was better than meniscal repairs in intact ACL.

## Conclusion

Our study concluded that arthroscopic meniscal repair is an effective way in the management of meniscal tears regarding clinical and functional outcomes.

## Conflict of interest

No conflict of interest was declared by all authors.
